# An initial investigation of serum cytokine levels in patients with gadolinium retention

**DOI:** 10.1590/0100-3984.2019.0075

**Published:** 2020

**Authors:** Holden T. Maecker, Weiqi Wang, Yael Rosenberg-Hasson, Richard C. Semelka, Joseph Hickey, Lorrin M. Koran

**Affiliations:** 1 Stanford University Medical Center, Stanford, CA, USA.; 2 Richard Semelka Consulting, PLLC, Chapel Hill, NC, USA.; 3 Hickey Wellness Center, Hilton Head, SC, USA.

**Keywords:** Cytokines, Pro-inflammatory, Gadolinium, Magnetic resonance imaging, Contrast-enhanced, Gadolinium deposition disease, Citocinas, Pró-inflamatório, Gadolínio, Ressonância magnética, Realce por contraste, Doença de deposição de gadolínio

## Abstract

**Objective:**

To determine whether individuals with proposed gadolinium deposition disease (GDD) have elevated serum levels of pro-inflammatory and pro-fibrotic cytokines, and whether specific cytokines are correlated with certain symptoms.

**Materials and Methods:**

Twenty-four participants recruited between May 2016 and June 2017 met GDD diagnostic criteria. The 64 control subjects provided serum samples before prophylactic flu vaccination. Serum cytokine levels were obtained with Luminex serum cytokine assay using eBiosciences/Affymetrix human 62-plex kits. Wilcoxon rank-sum tests were performed to assess the difference between the median fluorescence intensity values for the participants and the control group. Generalized linear models were built to evaluate the association between each cytokine of interest and selected participant symptoms.

**Results:**

Serum levels of 14 cytokines, including nine pro-inflammatory cytokines, were statistically significantly elevated compared to controls (*p* ≤ 0.05). Hypotheses regarding pro-fibrotic cytokines and cytokine links to specific symptoms' intensity were not confirmed.

**Conclusion:**

The statistically significantly elevated cytokines may be markers of susceptibility to GDD or agents of symptom induction. These findings suggest that individuals developing symptoms characteristic of GDD after a contrast-assisted magnetic resonance imaging should be studied to investigate whether gadolinium retention and elevated cytokines may be related to their symptoms.

## Abstract (pt)

**Objetivo:**

Determinar se indivíduos com doença de deposição de gadolínio (DDG) apresentam níveis séricos elevados de citocinas pró-inflamatórias e pró-fibróticas e se citocinas específicas estão correlacionadas com determinados sintomas.

**Materiais e Métodos:**

Vinte e quatro participantes recrutados entre maio/2016 e junho/2017 cumpriram os critérios de diagnóstico de DDG. Amostras de soro de 64 indivíduos controles foram obtidas antes de vacinação profilática contra a gripe. Os níveis de citocinas séricas foram mensurados com o ensaio Luminex usando *kits* 62-plex humanos. Foram realizados testes de Wilcoxon para avaliar a diferença dos valores médios de intensidade de fluorescência entre os participantes e o grupo controle. Foram construídos modelos lineares generalizados para avaliar a associação entre cada citocina de interesse e os sintomas dos participantes selecionados.

**Resultados:**

Níveis séricos de 14 citocinas, incluindo 9 citocinas pró-inflamatórias, foram estatisticamente significantes em comparação aos controles (*p* ≤ 0,05). Hipóteses sobre as citocinas pró-fibróticas e associação das citocinas com a intensidade de sintomas específicos não foram confirmadas.

**Conclusão:**

Citocinas estatisticamente elevadas podem ser marcadores de suscetibilidade para DDG ou agentes de indução de sintomas. Esses achados sugerem que indivíduos que desenvolvem sintomas da DDG após ressonância magnética com contraste devem ser estudados para investigar se a retenção de gadolínio e citocinas elevadas podem estar relacionadas aos seus sintomas.

## INTRODUCTION

A recently described disease termed gadolinium deposition disease (GDD) has been reported in patients with normal kidney function after magnetic resonance imaging (MRI) enhanced by gadolinium-based contrast agents (GBCAs)^(1)^. The symptoms begin unexpectedly within minutes (pain) to two months (skin thickening). The symptomatic patients exhibit gadolinium (Gd) retention a month or more post MRI, which is unexpected because contrast media half-lives are about 1.5 hours^(2)^. Gd retention in patients with normal renal function has been reported, albeit without concomitant investigation for retention-associated symptoms, in bone^(3)^, skin ^(4)^, cerebral spinal fluid ^(5)^, and brain ^(6,7)^.

We report the first study of *in vivo* cytokine serum levels in individuals meeting the proposed diagnostic criteria for GDD. The study's case-control design compared the serum cytokine levels of the study's volunteer sample to those of age and sex-matched controls. Based on *in vitro* and animal studies of Gd effects on cytokine expression, we hypothesized that serum levels of pro-inflammatory and pro-fibrotic cytokines would be abnormally elevated and associated with specific GDD symptoms, as detailed below.

In patients with markedly reduced renal function, exposure to GBCAs has caused a devastating illness, nephrogenic systemic fibrosis (NSF)^(1)^. Some characteristic NSF symptoms are common and chronic in GDD, including skin pain in a glove-and-stocking distribution with a burning or pins-and-needles quality, bone pain, distal leg and arm pain, spongy or rubbery subcutaneous skin thickening and tightness, and hyperpigmentation^(1,8)^. Many GDD patients, however, experience symptoms not reported in NSF, including frequent, persistent headaches, clouded mentation, and changes in vision or hearing^(9)^.

Many biological effects of Gd disrupt normal physiology and could lead to symptoms of toxicity^(10)^. The effect under study here is increased cytokine expression. Other disruptive effects include calcium channel blockade; inhibition of mitochondrial function; inhibition of Ca^2+^-activated enzymes; and, induction of oxidative stress.

*In vitro* studies report that both linear and macrocyclic GBCAs, as well as GdCl_3_, induce pro-inflammatory and profibrotic cytokine production by human monocytes^(11,12)^. The induced cytokines include the pro-inflammatory cytokines interleukin-6 (IL-6) and interferon-γ (IFN-γ) and the pro-fibrotic cytokines IL-4 and transforming growth factor-β (TGF-β), along with cytokine IL-13 (both fibrogenic and with mixed pro- and anti-inflammatory actions), and growth factor vascular endothelial growth factor (VEGF). The monocyte response variability across individuals suggests variability in individual sensitivity to GdCl_3_ and to the contrast agent studied, Gd-DTPA.

Rats injected with the GBCA gadodiamide, again reaching higher than clinical serum concentrations, exhibit significantly elevated serum levels of VEGF and the pro-inflammatory cytokines tumor necrosis factor-α (TNF-α), and monocyte chemotactic proteins 1 and 3 (MCP-1 and MCP-3)^(13)^.

Animal studies indicate that TNF-α can enhance pain sensation by increasing the sensitivity of peripheral nociceptive and dorsal root ganglion (DRG) neurons^(14-16)^. DRG inflammation up-regulates several pro-inflammatory cytokines including IL-6 and induces hyperalgesia/allodynia^(^17^)^.

Given these *in vitro* and animal study results and the symptoms of GDD, we hypothesized that serum levels of the pro-inflammatory cytokines IL-6 and TNF-α, of VEGF, and of the pro-fibrotic cytokines IL-4 and TGF-β will be higher in GDD patients than in age- and sex-matched controls. Secondarily, we hypothesized that serum levels of cytokines heretofore unstudied in relationship to Gd, both pro-inflammatory cytokines related to pain symptoms and pro-fibrotic cytokines related to skin thickening and other symptoms, will be higher in GDD patients than in controls.

## MATERIALS AND METHODS

### Recruitment

This case-control study enrolling a volunteer sample was approved by the Stanford Institutional Review Board. All participants signed written informed consent. GDD participants were recruited between May 2016, when the first participant was seen clinically, and June 2017. The subsequent participants were recruited via a study notice posted on the website https://gadoliniumtoxicity.com. One investigator (LMK) carefully interviewed all respondents by telephone to determine their study participation interest and their inclusion/exclusion criteria status.

Study inclusion criteria were: i) meets provisional diagnostic criteria for GDD, as specified in an FDA-approved protocol for treating GDD with DTPA chelation (Semelka and Jay, Investigational New Drug Application, 2016). These criteria are: presence of ≥ 3 of 8 symptoms-cognitive disturbance, extremity pain, arthralgia, chest wall pain, skin pain, headache, skin induration, and skin hyperpigmentation. Although the latter two symptoms were reported in investigator's interview and were not investigator-observed, excluding these symptoms did not affect participant eligibility. All study participants reported presence of at least three of the remaining six GDD symptom criteria; ii) had an unprovoked 24-hour Gd urine excretion level exceeding the laboratory norm ≥ 28 days after the symptom-inducing MRI.

Study exclusion criteria were: i) GDD present for > 2 years before the interview date (to limit possible biological variability associated with length of Gd exposure); ii) original GDD symptoms no longer causing substantial distress or impaired functioning in activities of daily living; iii) presence of an illness or medication known to markedly elevate serum cytokine levels, e.g., rheumatoid arthritis, systemic lupus, or cancer. Presence/absence was determined in the detailed clinical interview.

### Patient and control group demographic and clinical characteristics

Of the 48 respondents, 24 (50%) met the inclusion criteria and agreed to participate; 7 (15%) did not meet diagnostic criteria; 6 (13%) had been ill for more than two years; 4 (8%) had a confounding illness (cancer in 3, Lyme disease in 1); 3 (6%) were no longer suffering substantial distress or impaired functioning; 3 (6%) declined to participate after the study was explained; and 1 (2%) was too ill with GDD symptoms to participate. One participant (number 2) had been ill for 2.9 years, but was included before the 2-year cutoff criterion was decided upon. (None of the cytokine results for this subject were at the extremes for the participant group.)

The 24 participants, 20 women and 4 men, had a median age of 46 years (range, 27 to 82 years). Their GDD symptoms are shown in Table 1. Symptoms began on the day of the MRI for 13 participants, day 1 post-MRI for five, day 2 for two, day 3 for three, and day 14 for one. All participants quickly experienced new onset pain and cognitive problems and two-thirds developed new onset, frequent headaches.

**Table 1:** Frequency of GDD symptoms at onset and at time of blood draw.

GDD symptom	Frequency at GDD onset, n (%)	Frequency at time of blood draw, n (%)
Cognitive complaint	24 (100%)	22 (92%)
Extremity pain	19 (79%)	*
New, frequent headache	16 (67%)	20 (83%)
Chest wall pain	11 (46%)	*
Skin pain	18 (75%)	14 (58%)
Joint pain	18 (75%)	18 (75%)
Skin thickening	1 (4%)	16 (67%)
Skin hyperpigmentation	9 (38%)	12 (50%)
Bone pain	*	15 (63%)
Muscle pain	*	14 (58%)
Tingling sensations	*	24 (100%)
Muscle twitching	*	18 (75%)
Fatigue	*	22 (92%)
Dry eyes	*	17 (71%)
Eye pain	*	11 (46%)
Diminished visual acuity	*	16 (67%)

* Symptom not inquired about.

The 64 control subjects were 45 women and 19 men with a median age of 41.5 years (mean, 50.6 years; range, 26 to 88 years). The control group was drawn to closely match the GDD participants' demographic characteristics. None were acutely ill; their serum samples were obtained prior to a prophylactic flu vaccination. The control group was selected from several influenza vaccine studies (Brodin et al.^(18)^ and unpublished studies in which one author [HTM] collaborated) to provide a large enough, age- and sex-matched group whose cytokine results should adequately represent the cytokine range for healthy subjects. We cannot guarantee that there are no confounding factors in any of these individuals, but this control group is relatively large. Potential control subjects were excluded for a history of autoimmune disease, cancer (other than non-melanoma skin cancer), or other major chronic illness, but not for history of cardiovascular disease. Further description of the inclusion/exclusion criteria, the serum sampling schedule, and the assays utilized for all members of the control group is provided elsewhere^(18)^.

### Serum samples

The participants' blood samples were drawn by a visiting phlebotomist or at a local medical facility, rapidly spun down, and the serum frozen. Frozen serum was sent to the Human Immunology Monitoring Center at Stanford Medical Center. Participants' samples were compared with the samples from normal control subjects. Five participants provided serum samples before and after chelation treatment, having received Ca-DTPA on day 1 and Zn-DTPA on day 2. Serum cytokine levels before and one day after chelation were compared among the five participants. These samples were drawn 2.5 months, 6 months (n = 2), 11 months and 21 months after the symptom-inducing MRIs.

### Cytokine assay method

Luminex serum cytokine assay was performed by the Human Immune Monitoring Center. Human 62-plex kits purchased from eBiosciences/Affymetrix were used according to the manufacturer's recommendations with modifications as described elsewhere^(18)^. Each sample was measured in duplicate.

### Statistical methods

The cytokine median fluorescence intensity (MFI) values were normalized before being entered into the analysis. The samples were contained in four plates, so normalization to a shared control serum was used to minimize plate-to-plate differences. Control serum values (one value per cytokine) in one plate were taken as the reference objects, and the other plates were normalized according to their control serum value ratios (again, one ratio per cytokine) to remove the batch effects across plates.

Blank ratings in the symptom questionnaire (Table 1) were treated as zeros. Due to the non-normal distribution of the cytokine MFI values, Wilcoxon rank-sum tests were performed using stats package in R to assess the difference between the MFI values for the participants and the control group. For non-hypothesized cytokines, correction for multiple comparisons was made using Benjamini-Hochberg procedure.

Generalized linear models were built using stats package in R to evaluate the association between each cytokine of interest and selected participant symptoms, according to our *a priori* knowledge; therefore there was no adjustment for multiplicity testing. Analyses were conducted first without and then with adjustment for participant age, gender and body mass index. The tests were performed in JMP^®^ Pro 13.1.0 (SAS Institute Inc.) and R (version 3.4.3). All *p* values reported are two-sided; and a *p* value ≤ 0.05 was considered statistically significant.

## RESULTS

None of the indications for contrast-enhanced MRIs involved conditions with major effects on cytokines (Table 2). Some participants, however, had comorbid conditions or were taking medications or supplements that we learned, after the participants' cytokine levels had been determined, could modestly affect TNF-α and IL-6 serum levels (Table 2). The condition/medication-related serum cytokine levels of these participants, however, were randomly spread throughout the quartiles of the pertinent box plot displays. Moreover, the conditions/supplements more often lowered than raised cytokine levels; conditions/supplements could have raised TNF-α levels in four participants, but lowered the levels in seven; and, they could have raised the IL-6 level in one participant, but lowered the level in eight. Thus, these scattered, potentially confounding influences are highly unlikely the source of the significantly elevated serum levels of these two cytokines in the participants versus the controls.

**Table 2:** Reasons for MRI, and comorbid conditions, medications and supplements that could affect cytokine levels.

Comorbid condition	
Irritable bowel syndrome (3)*	Major depression (3)*
Medication or supplement	
Antidepressant drug (3)* Aspirin or NSAID (3)* Ashwagandha Astaxanthin	Lithium Milk thistle (2)* N-acetylcysteine (3)*
Reason for MRI	
Adrenal gland density Acoustic neuroma (follow-up) Brain scan for muscle pain and spasms Pituitary tumor (acromegaly) Vertigo Carotid bruit Chronic chest pain Cyst (ovarian, pancreatic, or parotid) Headaches (4)* Hernia repair mesh visualization	Injected facial fat Liver lesions (adenomas) Lower extremity pain Optic nerve scan Pulsatile tinnitus (2)* Pressure sensation behind eye Rule out cancer (cancer ruled out) Spine problems (5)* Thyroid enlargement Uterine fibroids

* Numbers in parentheses are the number of study participants with the comorbid condition, number taking the medication or supplement, or number having undergone an MRI for the reason indicated.

### GBCAs associated with symptom onset

Macrocyclic GBCAs were associated with symptom onset in 14 participants and linear GBCAs in 10 (Table 3). The participants' lifetime number of contrast-enhanced MRIs were: 1 (n = 15), 2 (n = 3), 3 (n = 1), 4 (n = 2), and 8 or more (n = 3). MRI-associated symptoms consistent with GDD began on the day of the MRI in 13 participants, one day later in 5, two days later in 2, three days later in 3 and 14 days later in 1. Following GDD symptom onset, additional contrast-enhanced MRIs with the same GBCA were performed in two cases, after 1 day and after 11 days, respectively. Thus, the most recent MRI was the symptom-associated MRI, except in the two patients who received additional MRIs with the symptom-related MRI's GBCA one and 11 days later, respectively. Table 3 displays the GBCA or GBCAs to which each participant was exposed, the GBCA associated with symptom onset, the Gd amount excreted in 24-hour unprovoked urinary specimens obtained ≥ 28 days after the participant's last MRI, the number of days after this MRI the measure was obtained, and the number of days after the last MRI the study blood draw occurred. Linear regression analysis revealed no significant relationship between serum cytokine levels and the number of days between symptom onset and study blood draw.

**Table 3:** Contrast agents, urine Gd amount and assay timing, and time to study blood draw.

Participant	Contrast agent(s)*	24-hour urine Gd (μg)^†^	Days after last MRI	Days from last MRI to study blood draw
1	OP. E, G^‡^	82.0^||^	28	121
2	MV	3.9^||^	45	1076
3	MH	3.9^||^	32	196
4	OP	2.5^§^	59	723
5	G	250^¶^	42	487
6	MV. MH. OM. G^‡^	1.9^||^	127	535
7	MH	7.8^§^	28	57
8	D	3.0^||^	38	293
9	OP. MV. MH^‡^	2.6^||^	54	332
10	G	1.3^||^	87	154
11	G	26.0^§^	41	565
12	G	1.8^||^	105	450
13	MH	29.0^§^	30	378
14	G	5.8^||^	38	520
15	G	8.8^#^	57	265
16	OM	5.0^||^	90	695
17	D	3.5^||^	30	94
18	G	8.3^§^	34	128
19	G	1.0^||^	89	179
20	OP	180^¶^	22	652
21	G. MH^‡^	33.0^||^	28	71
22	G	3.9^§^	77	116
23	MH. G^‡^	1.7^||^	214	248
24	G. OP^‡^	22.0^||^	36	84

* Macrocyclic GBCAs: G, gadobutrol (Gadovist); D, gadoterate (Dotarem). Linear GBCAs: MH, gadobenate dimeglumine (MultiHance); MV, gadopentetate dimeglumine (Magnevist); OM, gadodiamide (Omniscan); OP, gadoversetamide (OptiMARK).

† Gd amounts were determined by inductively coupled plasma mass spectrometry (ICP-MS).

‡ GBCA associated with symptom onset.

|| Mayo Clinic Laboratory, norm = ≤ 0.4 µg/24 hours based on unprovoked urine samples of men and women in the Mayo Clinic population known not to have undergone a contrast-assisted MRI within four days of the urine collection. The norm represents the 95th percentile in this sample.

§ Doctor's Data, Inc., norm = ≤ 0.6 µg/24 hours for women, ≤ 1.0 for men, based on unprovoked urine samples from individuals asked to refrain from a contrast-assisted MRI for at least 48 hours before starting urine collection. The norms represent the 95th percentiles in this sample.

# Genova Diagnostics, norm = ≤ 0.019 µg/24 hours based on unprovoked urine samples of questionnaire-qualified healthy men and women volunteers whose status re contrast-assisted MRI was not determined. The norm represents the 95th percentile in this sample.

¶ Chelation-provoked urine sample.

### Outcome of hypothesis testing

**Figure 1:**
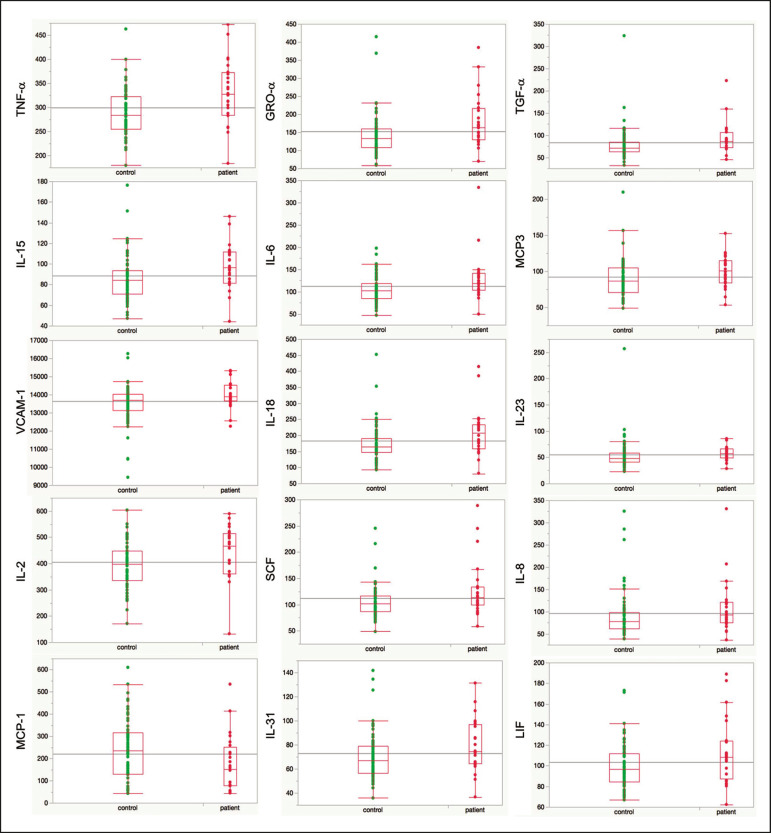
Scatter plots of the significantly elevated serum cytokines in GDD participants (red symbols) versus controls (green symbols). Y-axis is the median fluorescence intensity (MFI) of duplicate measurements for each subject. Gray bar representes the median of all participants. For each scattergram, the red line represents the median, boxes represent 1st-3rd quartile, and whiskers represent the 95% confidence interval for the distribution.

Serum levels of 14 of 62 tested cytokines were significantly elevated compared with those of the controls (Table 4, Figure 1). Only one, MCP-1 was significantly lower. For the five participants who received chelation treatment, the serum cytokine levels that were compared to control group levels were drawn before this treatment had begun. Adjusting the analyses for participant age, gender and body mass index did not change the results. The significantly elevated cytokines range in physiological effects, but these include prominent roles in inflammation and pain.

**Table 4:** Cytokines exhibiting significant differences in median fluorescence intensity between participants and controls.

Cytokine	Control Median (range)	Patient Median (range)	*P* value
TNF-α	284.0 (179.5-462.6)	327.0 (183.7-472.3)	0.004
GRO-α	134.1 (59.0-414.5)	163.9 (70.0-384.8)	0.006
TGF-α	71.5 (32.3-323.7)	85.8 (45.2-222.7)	0.01
IL-15	84.6 (47.2-176.3)	96.5 (44.3-146.0)	0.01
IL-6	101.7 (46.7-197.5)	118.9 (49.3-334.1)	0.01
MCP-3	86.5 (48.8-209.6)	101.1 (53.6-152.4)	0.02
VCAM-1*	13,705.9 (9,437.0-16,260.4)	13,915.4 (12,254.5-15,329.2)	0.02
IL-18	164.0 (92.4-452.1)	207.7 (80.9-414.3)	0.02
IL-23	48.0 (23.0-256.8)	57.5 (28.5-85.4)	0.02
IL-2	396.8 (171.5-603.4)	466.2 (132.5-589.6)	0.03
SCF*	101.7 (48.5-245.3)	113.8 (58.7-288.6)	0.04
IL-8	77.6 (38.4-325.5)	92.9 (36.1-331.0)	0.04
MCP-1	236.0 (42.8-610.3)	152.4 (44.6-534.6)	0.04
IL-31	66.8 (35.8-141.8)	74.4 (36.7-131.3)	0.05
LIF	96.6 (66.9-173.0)	108.5 (62.4-188.9)	0.05

* Cytokines that were not encompassed in our hypotheses.

Of the five cytokines found elevated after *in vitro* monocyte exposure to Gd^(9)^, only one, IL-6, was significantly elevated in the participants compared with the controls. One participant had a comorbid condition that tends to increase IL-6 serum levels and eight were taking medications/supplements that tend to lower these levels. Serum levels of the other cytokines-VEGF, IL-4, IFN-γ and TGF-β, which are involved in angiogenesis, fibrosis, wound repair, cell growth, cell proliferation and stimulating macrophage phagocytosis-were not significantly elevated in the participants' serum.

Serum level of the pro-inflammatory cytokine TNF-α was also significantly elevated. Four participants had conditions that tend to increase TNF-α serum levels and seven were taking medications/supplements that tend to lower these levels.

As hypothesized, pro-inflammatory and inflammation-related cytokines previously unstudied in relationship to Gd exhibited significantly increased serum levels: IL-8, IL-18, IL-23, IL-31, TGF-α, growth related oncogene alpha (GRO-α), and leukemia inhibitory factor (LIF). Though not specified in our hypothesis, serum MCP-3 levels were also elevated. The elevations of vascular cell adhesion molecule (VCAM), stem cell factor (SCF) and IL-15, which stimulates natural killer cells to kill viruses, were unexpected.

The hypotheses regarding associations of specific individual cytokines with the pain symptom cluster or with individual symptoms were not confirmed. No symptom or symptom cluster yielded a *p* value of < 0.05. However, higher serum levels of IL-10 were significantly associated with reduced muscle pain ratings (*p* = 0.005).

### Chelation effects on cytokine levels

**Figure 2:**
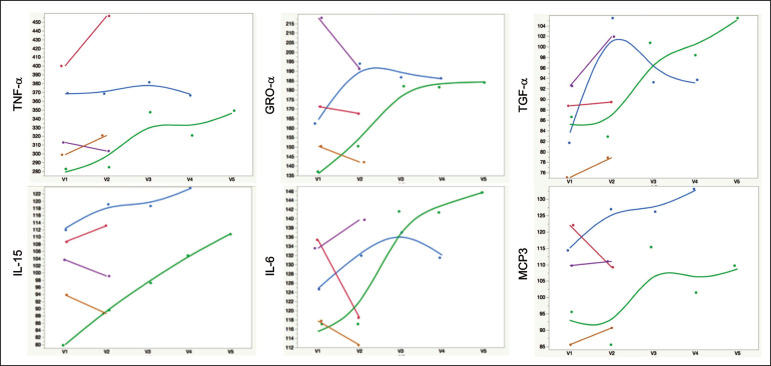
Longitudinal trends in serum cytokine MFI for GDD participants who provided samples before and after chelation treatments.

For the serum samples from the five participants providing samples before and after one or more chelation treatments with DTPA, we focused on the six cytokines most significantly elevated in the whole groups' samples compared to the controls: TNF-α, GRO-α, TGF-α, IL-15, IL-6, and MCP-3. Of these cytokines, five participate in inflammatory response, and one, IL-15, activates cell proliferation and natural killer cells. In general, post-chelation increases in these cytokine levels were more frequent and larger in participants whose serum was drawn sooner after their last MRI. More specifically, cytokine level increases for IL-6 were larger for all three participants drawn sooner than for those drawn at 11 and 21 months post-MRI. One participant demonstrated an increase in all six cytokines post-chelation, one in five of the six, one in four, and two in two. Looked at together, the patterns of cytokine changes post-chelation were patient-specific, but with many cytokines following a similar pattern in a given patient, often increasing immediately after chelation (Figure 2).

## DISCUSSION

This first study of cytokine serum levels in individuals meeting proposed diagnostic criteria for GDD after exposure to linear or macrocyclic GBCAs found significantly elevated serum levels of multiple pro-inflammatory cytokines, but not of pro-fibrotic cytokines, as compared to the levels in normal controls. These cytokine findings are the first biological abnormalities reported in this disorder. We intentionally used a multiplex assay to assess a large battery of cytokines in order to discover possible changes beyond those we hypothesized, which hypothesized changes were based on reported GDD symptoms and on the findings from the *in vitro* and animal studies^(11,13,19)^.

Given that the elevated cytokine levels were observed in subjects exposed to six different GBCAs, it is uncertain whether all GBCA agents had sufficiently similar immunologic effects or if some consistent de-chelated species of Gd was responsible (e.g., a Gd salt or Gd-protein macromolecule). No significant difference in cytokine levels was seen between participants receiving the most commonly represented GBCA (gadobutrol) and those receiving other GBCAs.

Because no serum samples were available from before exposure to the symptom-associated GBCAs, we cannot be certain that the elevations are related to Gd retention. However, the cytokine increases frequently seen after induction of Gd into the circulatory system via chelation, lend weight to the possible association. Perhaps the elevations signal patients who are more sensitive to cytokine stimulation effects of Gd, as proposed by the group that first described GDD^(10)^.

Second, the participant and control group cytokine distributions overlap. This is not unexpected, given the broad range of normal human variation^(20)^, and the findings in other disease settings such as chronic fatigue syndrome^(21)^, dilated cardiomyopathy^(22)^, and hepatitis C infection in abstinent alcoholics^(23)^.

Many factors, including obesity, presence of infectious disease, chronic stress, menstrual cycle day, diet, and time of day a serum sample is obtained, can influence the levels of pro-inflammatory cytokines. As noted, however, adjusting the analyses for body mass index did not change the results. Further, one study author (LMK) confirmed that no study participant had a cold, flu or other infectious disease when the serum sample was drawn. With regard to chronic stress, four of the nine abnormally elevated, pro-inflammatory cytokines, TNF-α, TGF-β, IL-2, and IL-6, observed in the participants have been reported elevated in chronic stress^(24)^. However, the four other pro-inflammatory cytokines elevated in chronic stress studies were not elevated in our participant sample, nor were five of the nine such cytokines observed in the sample. Thus, while chronic stress may have played a role in the observed pattern of elevated pro-inflammatory cytokines, the pattern is unlikely to be due solely to chronic stress.

The distributions of the other factors, menstrual cycle day, diet, and time of day of serum sample draw, would be expected to be similar within the participant group and the age- and sex-matched control group. The consistent pattern of elevated inflammatory cytokines in the participant group is unlikely to have occurred by chance affecting all these potential influences in one direction in the participant group and in the other direction in the control group.

The elevated cytokines we found have plausible mechanistic connections to the GDD symptoms of chronic pain and new, frequent headaches. IL-6 promotes increased levels of MCP-3, which in turn appears to play a role in neuropathic pain states^(25)^; and, the pain of GDD sufferers is frequently of neuropathic quality. The elevation of TNF-α is of interest in that it both plays a role in inflammation and increases the excitability of nociceptive neurons in the DRG^(26)^. The abnormal elevation of SCF, which stimulates melanocytes^(27)^, is of interest given that half the participants complained of increased skin pigmentation. The possible roles of all of these abnormally elevated cytokines in producing GDD symptoms deserve exploration in future research. The degree of participants' cytokine level elevation did not diminish with length of illness, which finding is consistent with the persistence of their GDD symptoms. Moreover, the adverse drug reaction probability scale^(28)^ indicates that Gd retention is at least a "probable" cause, i.e., the participants' symptom picture scores 7 of 13 points; "probable" is a score of 5 to 8 points. In addition, Gd chelation with DTPA can provoke transient symptom worsening, likely due to temporary exposure to increased circulating Gd^(27)^.

The non-confirmation of our hypotheses relating specific cytokines to a pain symptom cluster and other symptoms may reflect a true absence of relationships, the small sample size, clustering pain symptoms inappropriately, the use of an unvalidated questionnaire, or collecting serum samples at different times of day. The absence of an elevated serum level of IL-10, except in relationship to muscle pain, suggests this compensatory mechanism plays only a limited role in the body's chronic response to Gd physiological actions.

The reason for the cytokine level changes immediately after DTPA chelation is not clear. Suggested explanations include that the transmetallation process converting Ca-DTPA to Gd-DTPA during chelation creates Gd exposure; reintroduction into the vascular system of Gd as Gd-DTPA or other Gd species that stimulates immune cells to produce cytokines; or cytokine response simply to Gd-DTPA itself^(29)^. The differences in the participants' cytokine responses to chelation also echo the variable responses seen in the *in vitro* study of monocyte preparations from healthy individuals^(19)^.

This study has several limitations. First, the sample size is small. Second, although patients receiving a GBCA injection but not reporting post-MRI symptoms would be an ideal control group, our approach, employing a control group of random, healthy subjects is a standard one in medical research, including prior research on Gd toxicity^(30)^. Third, the participants' cytokine levels were studied at varying times after symptom onset. As noted, however, serum cytokine levels were not significantly related to the time between symptom onset and serum draw. Fourth, the participants' pre-MRI cytokine levels were not ascertainable. Although participants' blood samples were not all drawn at the same time of day, this was equally true for the control subjects. And, while, some participants' comorbid condition, medications or supplements could affect certain cytokine levels, these effects were more often to lower than to raise these levels. Future studies utilizing larger patient samples, designs able to test for causality, and more closely matched control groups, are needed.

In summary, GDD appears to be associated with elevations of cytokines that are pro-inflammatory or involved in inflammation; some of these cytokines can also enhance pain sensation, a common symptom in the disorder. These biological findings suggest that individuals with normal renal function who complain of symptoms characteristic of GDD after a contrast-assisted MRI should be tested to determine whether their symptoms may be associated with Gd retention. In such cases, preliminary evidence suggests chelation with DTPA may be beneficial. Whether and how symptoms of GDD are related to cytokine changes and the other biological effects of Gd observed *in vitro* and in animal studies deserve further investigation.
